# Therapeutic Potential of Mesenchymal Stem Cells in the Treatment of Epilepsy and Their Interaction with Antiseizure Medications

**DOI:** 10.3390/cells11244129

**Published:** 2022-12-19

**Authors:** Maryam Rahimi Tesiye, Mohammad Gol, Mohammad Rajabi Fadardi, Seyede Nasim Mousavi Kani, Anna-Maria Costa, Maryam Ghasemi-Kasman, Giuseppe Biagini

**Affiliations:** 1 Faculty of Life Science and Biotechnology, Shahid Beheshti University, Tehran 19839-69411, Iran; 2 Department of Biomedical, Metabolic and Neural Sciences, University of Modena and Reggio Emilia, 41125 Modena, Italy; 3 PhD School of Clinical and Experimental Medicine (CEM), University of Modena and Reggio Emilia, 41125 Modena, Italy; 4 Student Research Committee, Babol University of Medical Sciences, Babol 47176-47745, Iran; 5 Cellular and Molecular Biology Research Center, Health Research Institute, Babol University of Medical Sciences, Babol 47176-47745, Iran; 6 Department of Physiology, School of Medical Sciences, Babol University of Medical Sciences, Babol 47176-47745, Iran

**Keywords:** antiseizure medications, epilepsy, mesenchymal stem cells

## Abstract

Epilepsy is a life-threatening neurological disease that affects approximately 70 million people worldwide. Although the vast majority of patients may be successfully managed with currently used antiseizure medication (ASM), the search for alternative therapies is still necessary due to pharmacoresistance in about 30% of patients with epilepsy. Here, we review the effects of ASMs on stem cell treatment when they could be, as expected, co-administered. Indeed, it has been reported that ASMs produce significant effects on the differentiation and determination of stem cell fate. In addition, we discuss more recent findings on mesenchymal stem cells (MSCs) in pre-clinical and clinical investigations. In this regard, their ability to differentiate into various cell types, reach damaged tissues and produce and release biologically active molecules with immunomodulatory/anti-inflammatory and regenerative properties make them a high-potential therapeutic tool to address neuroinflammation in different neurological disorders, including epilepsy. Overall, the characteristics of MSCs to be genetically engineered, in order to replace dysfunctional elements with the aim of restoring normal tissue functioning, suggested that these cells could be good candidates for the treatment of epilepsy refractory to ASMs. Further research is required to understand the potential of stem cell treatment in epileptic patients and its interaction with ASMs.

## 1. Introduction

Epilepsy is a common neurological disorder, characterized by spontaneous recurrent seizures (SRSs), that affects approximately 1% of the population worldwide [1]. Approximately 65% of patients remain seizure free after undergoing treatment with antiseizure medications (ASMs) as a single administration or in combined therapy [2]. According to the mechanism of action, ASMs are classified into four groups: sodium (Na+) channel blockers, (γ-aminobutyric acid) GABA analogs, synaptic vesicle protein 2A binders, and others with multiple mechanisms [3]. Understanding the mechanisms of epilepsy is essential in establishing appropriate and valuable treatments [4]. Although pharmacological interventions effectively control SRSs in many patients, some patients also undergo surgery to remove brain-specific tissues. However, it should be considered that most epilepsy surgeries have been performed on the adult population with specific histopathologic features, and the long-term consequences of such surgeries are not usually devoid of unwanted consequences. Furthermore, in 7–35% of children, seizures recur with an increased duration of follow-up after epilepsy surgery [5].

Stem cell therapy might represent an alternative promising treatment for epilepsy, as suggested by the positive results that have emerged from animal models. Indeed, its beneficial effects could be related to the modulation of neural circuits, particularly regulation of aberrant hippocampal post-injury plasticity and release of neurotrophic and immune-modulating factors, which in turn prevent disease progression and improve cognitive function [6,7,8]. Moreover, the high therapeutic potential of stem cells, along with existing drug treatments, has been shown in a wide range of disease models. Various ASMs, such as sodium valproate (VPA), are used as priming molecules and pluripotency inducers of stem cells. VPA was initially discovered as an anticonvulsant, and is used today to treat a variety of seizure disorders as well as bipolar disorders (e.g., manic depressive illness) [9,10]. In the 1990s, Wilmott et al. used cell nuclear transfer to demonstrate that somatic cells could be reprogrammed into an undifferentiated embryonic state [11]. At that time, their attempts to produce such cells were unsuccessful, and further detailed studies were conducted in this field [12]. The first cell therapy study on epileptic animals was performed by Huber et al. in 2001. For this purpose, they used genetically modified fibroblasts to deliver adenosine encapsulated in a semipermeable polymer. The results showed that the incidence of epilepsy was reduced in kindled rats [13].

In general, stem cells are undifferentiated cells with a high capacity for proliferation and differentiation into various cell types; more importantly, they have self-renewal potential. This feature attracted a great interest in the treatment of various diseases, such as epilepsy [14]. Stem cells express an array of products, such as indoleamine 2-3 dioxygenase, prostaglandin E2 (PGE2), transforming growth factor β (TGFβ), other growth factors, anti-inflammatory or neuroprotective cytokines, and interleukins that affect how these cells interact with the host body and modulate the disease [15,16,17]. Other prominent properties include anti-apoptotic, antioxidant, and trophic effects that cause neural differentiation and modulate local or systemic immunity [18]. Evidence about their role in the treatment of epilepsy is growing, and there have been many successes in animal models. Moreover, the therapeutic potential of stem cells can be further improved by genetic engineering methods such as culture regimen adjustments, scaffold encapsulation, and genetic modification [19,20].

**Figure 1:**
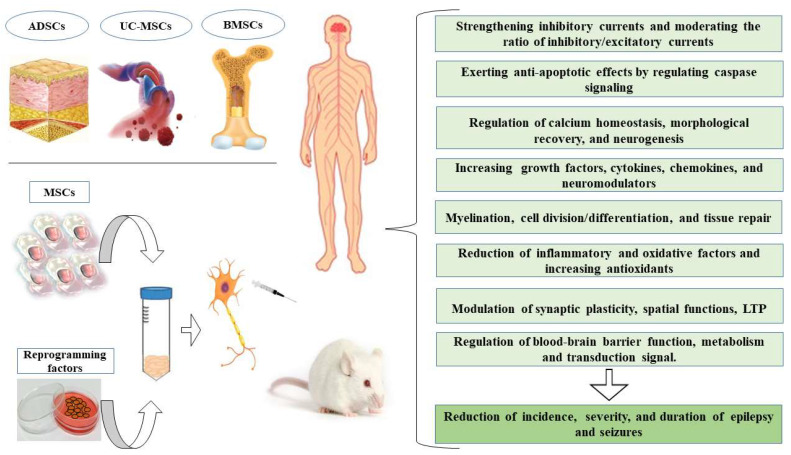
The potential beneficial effects of mesenchymal stem cells (MSCs) in the treatment of epilepsy. BMSCs, bone marrow mesenchymal stem cells; ADSCs, adipose-derived stem cells; UC-MSCs, umbilical cord-derive, LTP, long-term potentiation.

The goals of cell-based therapy in epilepsy (Figure 1) are to replace lost cells, reconstruct impaired brain circuits and use these cells as a carrier of endogenous neurotrophic compounds or as a vehicle to transfer ASMs. In fact, these cells migrate towards the chemotactic proteins secreted from the target tissue by expressing various types of cytokine and chemokine receptors; therefore, they can be suitable carriers for the transfer of therapeutic molecules [21,22]. Stem cells can be obtained from different regions of the body and are placed in various categories based on several parameters, but they are similar in many aspects (Table 1). Generally, they are divided into two categories, which include embryonic and adult stem cells [23]. In the following chapters, some of the possible mechanisms of ASMs in combination with mesenchymal stem cells (MSCs) to enhance their therapeutic properties will be investigated. Then, the special role of MSCs in the treatment of epilepsy will be discussed.

## 2. MSCs

MSCs, with their fibroblast-like morphology and specific marker expression, are intensively investigated for the treatment of neurological diseases. These cells are generally positive for CD105, CD29, CD73, CD90, and CD44, as thoroughly reviewed by Feng-Juan et al. in 2014 [46]. The primary sources of MSCs include bone marrow, cord blood and umbilical cord tissue, adipose tissue, synovial membrane, placenta, tooth pulp, periodontal ligament and the endometrium. These cells secrete multiple growth factors and cytokines under a paracrine or autocrine mechanism to afford endogenous protection or recovery responses. Furthermore, given their ability to migrate, they are capable of homing to the injured areas [47,48,49]. These cells encode genes that are involved in angiogenesis, immunity, and neural functions [50]. The low probability of developing teratomas, high homing potential, the production of neuroprotective cytokines such as amphiregulin, macrophage inflammatory protein-3 beta (MIP-3b), fibroblasts growth factor-6 (FGF-6), glucocorticoid-induced tumor necrosis factor receptor family-related gene (GITR), interleukin 10 (IL-10), osteoprotegerin, and neurotrophins like brain-derived neurotrophic factor (BDNF), along with their immunomodulatory and anti-inflammatory properties, make MSCs good candidates for cell therapy [51,52].

Like other stem cells, MSCs can be genetically modified and programmed to deliver various therapeutic factors such as nerve growth factor (NGF), glial cell line-derived neurotrophic factor (GDNF) and BDNF that is involved in tissue repair and growth [53,54]. The MSCs are derived from various sources in the body (Table 1) and in the following subheadings, we will examine their effects on epilepsy treatment.

### 2.1. Umbilical Cord-Derived Mesenchymal Stem Cells (UC-MSCs)

It has been well documented that human MSCs derived from Wharton’s jelly of the umbilical cord (hUC-MSCs) have therapeutic uses after childbirth. These cells are abundant and accessible resources that are easily prepared, stored, and transported and have no risk of transplant rejection or ethical problems. Due to their multi-directional differentiation abilities, self-renewal properties, low immunogenicity, and immunosuppressive potential, in a conditioned environment, hUC-MSCs can differentiate into glial or neural cells [55,56,57].

In temporal lobe epilepsy (TLE) with hippocampal sclerosis, the size of the hippocampus is significantly reduced due to pyramidal and GABAergic neuronal loss in the Cornu Ammonis 1/3 (CA1/CA3) regions. Depending on the environment in which hUC-MSCs are grafted, they release various cytokines, including MIP-3b, FGF-6, GITR, amphiregulin, and osteoprotegerin. All of these factors are involved in neural growth and have anti-inflammatory effects that attenuate the disease. In the rat pilocarpine model of TLE, it was shown that bilateral transplantation of hUC-MSCs into the hippocampus reduces neurological damage by replacing neurons, stimulating neurogenesis, and producing neuroprotective factors. Animals showed longer latencies to epilepsy onset, shorter seizure frequency and duration, and no convulsive activity was observed up to one month later. These results suggest that cytokines released from transplanted hUC-MSCs are able to modify the course of epileptogenesis [58] (Table 2 and Table 3). The same results were obtained in another study using human umbilical cord blood stem cells (hUCBC), which indicates that early interventions can protect the brain against the establishment of epilepsy [59].

The therapeutic potential of hUCBC has also been evaluated for patients suffering from Dravet syndrome (DS), which is a drug resistant and severe form of epilepsy in children. In this study, the effect of hUC-MSCs on a human induced pluripotent stem cell model of DS was evaluated. According to the results, hUC-MSCs decreased the levels of intercellular calcium (Ca2+), malondialdehyde (MDA), and reactive oxygen species (ROS) by increasing the levels and activity of various antioxidant enzymes such as superoxide dismutase1/2 (SOD1/2), glutathione (GSH), and glutathione peroxidase (GPX). The levels of anti-inflammatory factors (IL-10/6, and TGF-β) were also significantly increased while the expression of interleukin-1β and tumor necrosis factor-α (TNF-α) were reduced. This indicates that these cells have antioxidant and anti-inflammatory properties, and are able to counteract mechanisms that play an important role in the development of epilepsy [85]. 

In the rat model of pentylenetetrazole (PTZ)-induced seizure, it was found that PTZ, by competing with GABA receptors, induces excessive calcium influx and disrupts cell function. These impairments are associated with mitochondrial damage followed by oxidative stress. The anticonvulsant effects of hUC-MSCs appear to be through the reversal of these processes, possibly by increasing the expression of neuronal and glial markers, which themselves induce restorative effects. This study verified the therapeutic potential of hUC-MSCs in seizure control, motor and cognitive impairment, and GABAergic neurotransmission disruption [60]. Another study evaluated the efficacy of hUC-MSCs transplantation in chronic epileptic rats, and revealed that unilateral intra-hippocampal transplantation was followed by cell migration to the opposite hippocampus. Despite the fact that transplanted cells survived, they did not differentiate into specific neural subtypes and were unable to repair neural circuits in this study. They partially, however, restore the impaired glucose metabolism in both injected and contralateral areas, and this metabolism remained stable after eight weeks. As a result, they exerted a therapeutic effect by preventing the progressive hypometabolism in the epileptic regions, thus suggesting a possible promising effect of hUC-MSCs on the epileptic networks [61].

Oxidative stress-mediated neuronal damage is a hallmark of many neurological disorders, such as seizures. Previous findings have shown that MSCs and MSC-derived extracellular vesicles (EVs) can inhibit oxidative injury. Increasing evidence suggests that EVs are more effective than their parental cells (e.g., MSCs) as restorative therapy in neurological disorders. This feature seems to be due to the existence of functional cargoes in EVs that play roles in metabolic regulation, immunomodulation, and signal transduction. Additionally, EVs have drawn a lot of attention due to their ability to cross the blood–brain barrier (BBB) and their resistance to freezing and thawing. EVs contain various ribonucleic acids (RNAs), proteins and lipids that are involved in the modulation of antioxidant activities. In animals treated with MSC-EVs, levels of oxidative markers such as 8-hydroxy-2’-deoxyguanosine, dityrosine (protein oxidative marker), and 4-hydroxy-2-nonenal were reduced in spite of the similar seizures experienced by rats belonging to the different groups of treatment. A possible mechanism involved in these effects was the activation of nuclear factor erythroid 2–related factor 2 (Nrf2) signaling pathway. Improving the properties and excitability of nerve membranes and subsequently repairing hippocampal neural circuits in animal models demonstrated the high efficacy of MSC-EVs in the treatment of epilepsy. In addition, in pyramidal neurons of the CA1 region, spine density decline, dendritic complexity and mitochondrial dysfunction were recovered, and glutamate receptor gene expression patterns were changed by EVs treatment, implying the reconstructive properties of MSC-EVs on seizure-induced morphological alterations [62].

### 2.2. Bone Marrow Mesenchymal Stem Cells (BMSCs) 

The anti-seizure effects of BMSCs are mainly due to increasing GABAergic neurons and upregulation of glutamate decarboxylase 67 (GAD67) + in inhibitory neurons of the CA1 and dentate hilus. BMSCs can also prevent mossy fiber sprouting (MFS) and increase the expression of anti-inflammatory cytokines, in presence of reduced neurological defects. It was shown that transplanted BMSCs decrease the concentration of the inflammatory enzyme myeloperoxidase in the hippocampus, which in turn induces a considerable reduction in the duration and frequency of seizures, GABAergic and inhibitory pyramidal neuron loss, and brain edema. In addition, data from behavioral tests revealed improvements in spatial performance and learning. It should be noted that such improvements were achieved in the short-term analysis of subjects rather than in a long-term study [63,64]. The same results were obtained by transplanting medial ganglionic eminence GABAergic precursors to the hippocampus in the epileptic rats [88].

In another study, the effects of intrahippocampal injection of BMSCs, which were manipulated to release IL-13, have been evaluated by measuring hippocampal paroxysmal discharges in mice receiving this treatment. According to the results, although the amount of discharge decreased, no neuroprotection was obtained. The number of macrophages, microglia, or astrocytes around affected areas was not significantly increased significantly. Such an effect may be due to the fact that IL-13 was not released from transplanted cells as expected, so it requires a more detailed investigation [65].

Given that disruption of adenosine A1 receptors (A1Rs) or disruption of the adenosinergic system is associated with chronic seizure activity, it has been suggested that stem cells engineered to release adenosine or to express adenosine receptors could be a valuable treatment. Adenosine mainly acts through A1Rs and A2a receptors (A2aRs). While A1R receptors are reduced in epilepsy, A2aRs are sharply upregulated. However, BMSCs transplantation caused considerable upregulation of A1R expression and downregulation of A2aR, showing that they can reverse the imbalance of adenosine receptors and improve the outcome of disease [66,67]. The same results were obtained from the mouse model of focal CA3 spontaneous seizures. As it showed, seizure intensity was considerably reduced after hippocampal injection of human hMSCs engineered to release adenosine [68].

Interestingly, down regulation of adenosine kinase (ADK), the main adenosine removing enzyme, in hMSCs by lentiviral expression of a particular miRNA against ADK, has also been shown to reduce the severity of seizures and exert neuroprotective effects [69]. In general, most of BMSCs effects in the hosting body are mediated by secreting neuroprotective, neurogenetic, immunomodulating, and cell survival factors, such as insulin-like growth factor-1 (IGF-1), NGF-β, BDNF, hepatocyte growth factor (HGF), basic fibroblast growth factor (bFGF), TGF-β, GDNF, FGF-6, GITR, vascular endothelial growth factor (VEGF), and MIP-3β [8].

Due to the fact that epilepsy is associated with a significant reduction in the number of neurons and volume of the hippocampus, intravenous (IV) injection of BMSCs and their migration and establishment in the hippocampus can compensate the lesion size and restore the morphology and function of the hippocampus. Although the migration process occurred quickly, it was observed that the number of transferred cells was relatively small, and after four months, the grafted cells were distributed throughout the whole brain. Based on the findings, in rats receiving BMSCs, long term potentiation (LTP) was improved and a higher amplitude of fast excitatory post-synaptic potential was observed in the short term. This finding was interpreted as a protective and regenerative effect of BMSCs on hippocampal synaptic circuits. It should be noted that after a long-term analysis, one-quarter of the treated animals developed SRSs, although their duration and frequency were lower [70,71].

Implantation of BMSCs also caused a significant reduction in pro-inflammatory cytokines. It is well known that too much production of pro-inflammatory cytokines inhibits astrocytic glutamate uptake, reduces GABA levels, potentiates N-methyl-D-aspartate receptor function, and, consequently, causes Ca2+ influx and inhibits K+ efflux, thereby inducing neuronal hyperactivity and neurodegeneration. BMSCs significantly protected the hippocampal neurons and networks by inhibiting neuronal loss in the CA1, CA3, and dentate hilus regions. In addition, reactive gliosis was less intense in treated groups than in the control group [72].

It is reported that BMSCs express specific GABAergic neuron markers such as GAD67 and, obviously, the same general neuronal marker neuron-specific nuclear protein. Indeed, these immature cells differentiate into GABAergic neurons and thus increase inhibitory transmissions. Based on the findings, this ability was significantly increased by silencing the hairy and enhancer of split-1 (HES1) signaling, which is a member of the basic helix–loop–helix transcriptional repressors that control cell division and differentiation during neurogenesis in BMSCs. After such a knockdown, the proliferation of BMSCs was reduced. However, after switching off HES1 signaling in rats receiving BMSCs, the mortality rate of animals due to seizures and electroencephalogram (EEG) amplitude showed a considerable reduction, which indicates a functional recovery in groups receiving such transplantation [73]. Another study showed that after transplantation HES1-downregulated BMSCs into the lateral ventricle of pilocarpine-induced epileptic rats; these cells migrated to adjacent parahippocampal areas, where they differentiated into GABAergic cells or astrocytes. Then, by amplifying the GABAergic transition, the BMSCs reduced the frequency and rate of SRS, and thus it seems that the BMSCs modification could increase their efficiency for treatment [74].

Bone marrow mononuclear cells (BMNCs), which are used in treatment of TLE, release various growth factors, including NGF, GDNF, VEGF, TGF-b1, and BDNF. Increased messenger RNA (mRNA) expression of BDNF and NGF in the hippocampus of rats a few days after BMNCs transplantation, with the reconstruction of neural networks, plays a role in the repair of damaged areas. GDNF, as a survival factor, prevents inflammation and controls neuronal excitability. Increased VEGF expression significantly modulates learning and memory after seizures and better preserved the cognitive performance. Conversely, TGF-b1 expression in neurons and astrocytes, which is involved in epileptic activity via BBB disruption, was decreased after BMNCs implantation [75].

The treatment of the PTZ model with BMSCs in a period of one to two months recovered the imbalance of excitatory/inhibitory neurotransmitters in the hippocampus, cerebral cortex, and cerebellum. Such treatment also reduced the levels of oxidative stress markers that impair BBB permeability, thus restoring its integrity. Furthermore, after BMSC transplantation into the epileptic brain, inflammatory markers such as TNF-α and IL-6 were significantly reduced. These effects seem to be due to increased neurogenesis, neuroprotection, and cytokine modulation promoted by growth and neurotrophic factors [76].

In this regard, another study on the role of oxidative stress in the incidence of epilepsy showed that ROS, by reducing the levels of antioxidants such as GSH and paraoxonase 1, and impairing calcium homeostasis and lipid peroxidation, lead to excessive excitability of the central nervous system (CNS). The ROS also induced apoptosis by modulating a number of factors, in particular B-cell lymphoma 2 (BCL2) associated X, apoptosis regulator, and caspase-3, and were associated with increased glutamate levels and seizure susceptibility. However, IV or intrahippocampal injection of BMSCs has been shown to reduce the incidence of epilepsy and its complications by diminishing oxidative stress and lipid peroxidation, and increasing antioxidant levels. BMSCs treatment also decreased the levels of excitatory amino acids, BDNF-mediated excite-toxicity, inflammatory factors such as IL-1β, TNF-α, apoptotic markers and caspase-3 in the hippocampus. In contrast, it increased anti-inflammatory factors such as IL-10 and TGF-β. Such changes shift cells to their normal state. In addition, they caused a remarkable reduction in immune-reactivity to IGF-1 receptors, caspase-3 and synaptophysin, and ameliorated the histological and neurochemical changes induced by pilocarpine. It should be noted that IC administration of BMSCs exerted more pronounced effects than IV injections [77].

Based on the results of another similar study, IV administration of BMSCs reduced cell death and caused the production of pivotal anti-inflammatory mediators. The administrated BMSCs also differentiated into neurons. However, their obvious function was to reduce the expression of inflammatory cytokines such as IL-6, TNF-a, and IL-1β while increasing the expression of anti-inflammatory cytokines such as IL-4, IL10 and nitric oxide synthase 2 (NOS2). Due to such effects, neurodegeneration was decreased in the CA1, CA3 and dentate hilus of hippocampus [78].

Another study confirmed that these cells exerted their effects via antioxidants factors such as erythropoietin and hemeoxygenase-1. During an epileptic seizure, elevated levels of cytokines like TNF-α activate microglial cells and cause upregulation of caspase-8 transcription. Caspase-8 then promotes tumor necrosis factor receptor 1 signaling and consequently stimulates apoptosis. However, transplantation of adipose tissue-derived MSCs (AD-MSCs) or BMSCs into epileptic rats resulted in a considerable minimization of caspase-8 levels in the brain and suppressed apoptosis. Additionally, the brain level of S100 calcium-binding protein β (S100β), a marker of BBB damage, decreased, and BBB recovery was mainly accomplished by potentiating TNF-α-stimulated gene protein-6 expression and the nuclear factor kappa-light-chain-enhancer of activated B cells (NF-κB) signaling pathway. Data analysis also revealed a close relationship between elevated heat shock protein 70 kilodaltons and Toll-like receptor-4 (TLR-4) genes expression and epilepsy. These factors are involved in oxidative stress and inflammation, respectively, and MSCs transplantation decreased both of them [79]. Moreover, intra-arterial delivery of autologous BMSCs releases paracrine factors into the host tissue, causing a compensatory response to changes in the extracellular environment of the host. Patients receiving this transplantation showed a better memory score and a reduction in the number of epileptic spikes [80]. An experiment in pilocarpine-treated rats also revealed that the animals had better performance in the Morris Water Maze (MWM) test, which indicates a long-term spatial memory and cognitive function improvement [81].

Additionally, intrathecal transplantation of BMNCs and BMSCs to drug-resistant epileptic patients was revealed to be workable and safe. This treatment was associated with reduced severity and incidence of epilepsy. It also changed the epilepsy types from polymorphic and tonic-clonic to less polymorphic and mostly tonic without secondary generalization. Thus, the bioelectric activity of the brain was improved. This study confirmed that the expression of various growth and nutritional factors such as HGF, NGF, BDNF, VEGF-A, ciliary neurotrophic factor, matrix metalloproteinase-2, and fibroblast growth factor 2 (FGF-2), which are involved in myelination, cell division, differentiation, and tissue repair, were increased [86].

Interestingly, changes in the levels of hormones such as follicle-stimulating hormone (FSH), luteinizing hormone (LH), triiodothyronine, thyroid-stimulating hormone, and estrogen have been shown to play a direct role in the development of epilepsy. It has been shown that epilepsy is related to a higher frequency of gonadotropin-releasing hormone secretion, which is followed by higher testosterone levels and FSH/LH ratios. Furthermore, increased activity and disruption in the hypothalamic–pituitary–adrenal (HPA) axis are involved in the development of epilepsy. It was shown that estrogen can increase the severity of cortical seizures induced by PTZ while also elevating the excitatory synapses and the number of dendritic spines in the hippocampal CA1 pyramidal neurons. It seems that psychosocial factors and ASMs are responsible for this alteration. However, in animals treated with BMSCs, the levels of estrogen and testosterone approached normal levels, and hypothesized that MSCs therapeutic effects are mostly due to their paracrine effects on endogenous cells, including the activation of restorative processes such as angiogenesis, neurogenesis, and synaptogenesis after stroke and neural injury [76].

### 2.3. Adipose-Derived Stem Cells (ADSCs)

Adipose-derived stem cells (ADSCs) are cells with a high ability to proliferate, regenerate, and differentiate into mesodermal and non-mesodermal cells. These cells can secrete cytokines, chemokines, and growth factors that cause them to act as paracrine tools. Furthermore, they exert anti-apoptotic, anti-inflammation, anti-fibrotic, and immunomodulatory effects [89,90].

In 2020, Szczepanik and colleagues [87] showed that in patients with mean age of 10 years and with refractory epilepsy who received an intrathecal infusion of autologous adipose-derived regenerative cells (ADRCs) (3 times every 3 months), all patients were in good condition, and no significant side effects were observed except for fever or pain in the area of liposuction after the procedure. During the next few months, some improvements in school, social functioning, and manual performance were observed in all patients. Like other MSCs, they released different chemokines, anti-inflammatory cytokines, modulators, and growth factors. However, the concentrations of five cytokines in the CSF, including IL-10, angiogenin 1, stromal cell-derived factor 1 (SDF-1) also known as C-X-C chemokine ligand 12 (CXCL 12), TNF-α and osteopontin significantly changed after transplantation. Among them, only the concentration of osteopontin as an inflammatory cytokine was reduced. TNF-α is known to play a role in inflammation and migration of transplanted cells. Angiogenin 1, like IL-10, has anti-inflammatory property in addition to angiogenesis. The SDF-1/CXCL12 signaling pathway is also involved in neuroplasticity, neurogenesis, and the regulation of GABA release. Overall, therapy with these cells in autoimmune refractory epilepsy seems to be safe and feasible [87].

Additionally, Hua He and colleagues evaluated the potential protective effects of ADSCs transplantation in the KA model of TLE in rats. Based on electroencephalogram recordings, after transplantation of ADSCs to rats, seizure activity was inhibited, and they had better performance in the MWM task. The molecular analysis also revealed that they secret various factors, such as neurotrophin 3 (NT3), BDNF, and NT4, which are involved in the suppression of apoptosis. This experiment showed that transplantation of human ADSCs has long-term protective effects against kainic acid-induced (KA) epilepsy in rats and improves the learning and memory of KA rats via releasing anti-apoptotic BDNF, NT3 and NT4 [82].

Another study showed that acute application of human ADSCs (hADSCs) 15 min before status epilepticus (SE) or during chronic stage of epilepsy in a pilocarpine mouse model caused an earlier reduction in seizure spike, BBB leakage, and inhibition of epilepsy development. Seven days’ treatment with hADSCs during the chronic phase of epilepsy (28th to 35th day after SE) suppressed or inhibited chronic SRS in the epileptic mice and decreased abnormal epileptic behavioral phenotypes such as cognitive impairment. The long-term hADSCs group that was injected with hADSCs every day for 7 days, performed better in the anxiety- and locomotion-related tasks than the epileptic control mice. Furthermore, long-term treatment with hADSCs exerted its effects through the paracrine expression of angiogenic or antiapoptotic factors, reducing BBB leakage and inducing revascularization mediated by bFGF and VEGF. The long-term effects of hASCs may be a result of the correction of the altered neuronal networks through decreased neuronal excitability and inflammatory responses. Moreover, the expression of the α-3 subunit of the GABA-A receptors was elevated in long-term treatment with ADSCs. This indicates that strengthening of inhibitory circuits is one of the therapeutic consequences of hADSCs [83].

Another successful ADSCs treatment was in a generalized tonic-clonic mouse seizure model, which was induced by maximum electroconvulsive shocks (65 mA, 60 Hz, 0.15 s duration). Transplantation of ADSCs into the hippocampus altered the seizure threshold and showed an anticonvulsant effect by protecting against tonic seizures or mortality and reduced the hippocampal expression of transcripts related to inflammatory responses such as IL-1β, IL-6, caspase-1, and iNOS, and increased the level of anti-inflammatory interleukin IL-4. One possible reason to explain the protection effect is that the transplanted cells showed a barrier effect, preventing the hyperexcitability in the local circuit and avoiding the spread (generalization) of seizures, expressed by reduction in duration (tonic) and in post-ictal period. These results suggest that the protective effects of MCAT cells are possibly promoted by the action of inhibitory factors and immunomodulatory mechanisms assigned to mesenchymal cells on seizure spreading [84].

## 3. Mechanisms for MSCs Enhanced Neuroprotection

The great ability of MSCs to protect against immune-mediated damage has been shown in different models. Neuroprotection and neurogenesis caused by stem cells should mainly be related to the release of growth factors such as BDNF, NGF, GDNF, VEGF, NT3, and platelet-derived growth factor (PDGF) [91]. Additionally, with the production of IGF-1, they seemed to play a role in the activation of neuroprotective signaling pathways such as PI3K/Akt and p44/42 MAPK and to reduce cell death caused by glutamate [92]. In animal models, it has been shown that MSCs could affect the function of brain cells such as microglia. They should not only able to prevent microglial cell proliferation but also prevent the release of pro-inflammatory factors from them. On the other hand, they seemed to induce the release of neuroprotective molecules from them and strengthen their phagocytosis ability [93].

## 4. Possible Mechanisms of Anti-Seizure Effects of MSCs-Based Therapy

MSCs might have potential to be an effective tool in epilepsy treatment, as they increase the GABAergic interneuron density within the hippocampus and inhibit mossy fiber sprouting. Thus, by increasing the release of inhibiting neurotransmitters, and by limiting the abnormal innervation of mossy fibers to the dentate inner molecular layer of the hippocampus, treatment with MSCs should limit the propagation of epileptiform activity and the development of seizures [94].

In a variety of animal models, it has been observed that grafting GABAergic precursor cells derived from fetal brain into epileptic foci could reduce seizure frequency. Furthermore, the precursor cells derived from the medial ganglionic eminence (MGE) of the embryonic brain displayed the most effective effect in limiting seizures. In this regard, MGE cells might be the most appropriate donor cell types for the treatment of epilepsy due to their ability to: (i) perform a pervasive migration; (ii) differentiate into different subclasses of GABAergic interneurons; (iii) integrate into synaptic connectivity; (iv) improve inhibitory neurotransmission in the hippocampus; and (v) markedly decrease the hyperexcitability and seizures in TLE [88,95,96,97,98]. Interestingly, human MGE-like (hMGE) cells generated from human-induced pluripotent stem cells (hiPSCs) that were grafted into the hippocampi of rats that developed SE resulted in a consistent reduction in the frequency and severity of SRSs. Moreover, when hMGE were integrated in the hippocampal circuitry and largely differentiated into GABAergic interneurons, their axons created synaptic connections on the soma and dendrites of the host granule cells of the dentate gyrus and CA1 pyramidal cells [99].

## 5. The Effect of ASMs on MSCs

There are numerous methods to facilitate the differentiation of stem cells into various cell lines, including exposing them to a variety of drugs and therapeutic molecules (Table 3 and Table 4). Indeed, priming molecules can be enriched in damaged tissues and act as stem cell chemo-attractants.

### 5.1. The Effect of Valproic Acid (VPA) on MSCs

Valproic acid (VPA) is an anticonvulsant agent for the treatment of epilepsy, as well as a mood stabilizer for the treatment of bipolar disorder. It is able to inhibit histone deacetylase (HDAC), which has been reported to play a role in the differentiation of mammalian cells [100,101].

The therapeutic effects of MSCs priming with cytokines, hypoxia, biomaterials, growth factors, and pharmacological factors have been shown in different studies. Primed MSCs potentiate neuroprotective effects mediated by autophagy in animal models of neurological diseases. In this regard, by regulating the expression of miRNAs that regulate autophagy and the release of exosomes, they are involved in increased cell survival [102]. Additionally, priming them with various cytokines, such as IFN-γ, IL-1β, IL-1α, and TNF-α can modulate MSCs’ immunosuppressive effects [103]. However, there is evidence that high doses of VPA, as well as long-term treatment, induce Parkinson’s symptoms in some patients, and these symptoms return to normal with dose reduction [104,105,106]. A study on pregnant women who used ASMs such as VPA, lamotrigine, carbamazepine, and phenytoin showed that the child’s brain development was disturbed and their IQ score was somewhat lower. In some cases, developmental problems or neurological disorders have also been reported [107,108,109].

The priming of MSCs in combination with low doses of VPA potentiated the self-renewal, migration, and anti-inflammatory effects of MSCs. VPA (2.5 mM for 3 h), in combination with lithium (2.5 mM for 24 h), caused a synergistic effect on MSCs’ migration and priming ability, and a neuroprotective effect against neural excitotoxicity [110,111] (Table 4). Moreover, it significantly increased the expression of C-X-C chemokine receptor type 4 (CXCR4) and its signaling, which subsequently affected the migration ability of MSCs. Further studies observed that the pretreatment of MSCs with VPA (2.5 mM for 24 h) could increase the expression of cytokine receptors such as CXCR7, thus affecting their efficiency [112]. The study on MSCs derived from cord blood also showed similar results [113], furthermore, the migration of these cells was influenced by the stromal SDF-1/CXCR4 and SDF-1/CXCR7 axis. Then, it was shown that when MSCs derived from cord blood were subjected to different concentrations of VPA (1, 2.5, 5, or 10 mM) for 3 h, their division rate was increased. However, in higher concentrations (10 mM), the opposite results were observed. These studies showed that epigenetic mechanisms might play an important role in the differentiation and division of MSCs, after VPA treatment. In addition to this, it was reported that MSCs treated with VPA (200 µg/mL for 12 h) increased the expression of proteins, such as Krev/rap1 interaction trapped-1 (KRIT1), which prevent the accumulation of oxidants inside the cell and inhibit oxidative stress [114].

**Table 4:** The effect of anti-seizure medications (ASMs) on mesenchymal stem cells (MSCs).

Drugs	The Source of MSCs	Genes and Signaling Pathways Involved	Ref.
Valproic acid	Cryopreserved rat MSCs	Increased cell migration ability	[110]
	Human umbilical cord-derived MSCs	Enhancement of cell migration and potentiating anti-inflammatory and immunity pathways	[111]
	Human bone marrow-derived MSCs	Increased function of stem cells by increasing the expression of CXCR7	[112]
	Cord blood mesenchymal stromal cells	Increased cell migration according to increased activity of SDF1/CXCR4 and SDF-1/CXCR7 signaling pathways	[113]
	Human bone marrow-mesenchymal stromal cells	Increased expression of KRIT1 and prevention of the accumulation of intracellular oxidants and reduction of oxidative stress	[114]
	Human umbilical cord-derived MSCs	Increased expression of mesenchymal and endodermal genes related to hepatic tissue/strengthening of CXCR4 signaling	[115]
	Human MSCs derived from adipose tissue	Increased MSCs migration ability by activating the CXCR4 signaling pathway	[116]
	Human bone marrow-derived mesenchymal stromal cells	Activation of CXCR4 signaling pathway and increased MSCs immortality	[117]
	Human bone marrow-derived MSCs	Increased expression of liver markers such as ALB, AFP, CK-18, TAT and increasing the differentiation of MSCs into liver cells	[118]
	Human umbilical cord-derived MSCs	Regulation of the expression of a group of miRNAs and hepatic differentiation	[119]
	Adipose tissue and bone marrow-derived MSCs	Increased expression of osteogenic genes such as RUNX, BMP2, p21WAF1, osterix, and osteopontin	[9]
	Tonsil-derived MSCs	Increased bone differentiation with CCN1 protein expression	[120]
	MSCs derived from pancreatic islets	Increased expression of genes related to beta cell neogenesis, such as NKX6.1/increase in the number of insulin-positive cells	[121]
	Mouse lip derived-MSCs	Increased expression of cardiac structural genes such as Cx43, βMHC, cTnI, and MLC2v and cardiac primary transcription genes such as NKX2.5, HAND2, HAND1, and GATA4	[122]
	Human Wharton’s jelly MSCs	Increased expression of neuronal markers such as Nestin, Neuro-D1	[123]
	Human bone marrow-derived mesenchymal stromal cells	Increased expression of neuronal markers such as GFAP, Musashi, CD133, Nestin-1, MAP-2, and KCNH2/5	[124]
	Human bone marrow-derived MSCs	Increased expression of markers of mature neurons such as Th, VAChT and Htr2a/decreased expression of oligodendrocyte and primary neurons precursors	[125]
	Mammary fat tissue and cord blood MSCs	Cell cycle inhibition in G2/M phase/enhancement of p21CIP1/WAF1 signaling pathway activity and cell cycle inhibition	[126]
	Mouse MSCs derived from bone marrow	Regulation of the expression of genes involved in energy metabolism such as PGC-1α, Cox6b2, and Atp12a and genes involved in antioxidant defense such as Serpinb1b, Gpx6, and Mt2/increased expression of genes involved in cell stress pathway such as Hsp27, Hox, and Hsp A1l, and anti-apoptotic pathways such as Erc1, Naip1, and Faim2/elevated expression of growth and trophic factors such as FGF-15, FGF-21, NDNF, GDF-1, BMP-3, and NTF3	[127]
	Human bone marrow-derived MSCs	Apoptosis induction and antiproliferative effects in T cells	[128]
	Cryopreserved rat MSC	Enhancement of CXCR4 signaling/increased expression of mesenchymal markers such as fibronectin and CD54	[129]
	Human bone marrow-derived MSCs	Increased expression of tumor suppressor genes such as Cx43 and Cx26 and induction of apoptosis	[130]
Phenytoin	Dental pulp-derived-MSCs Mouse lip derived-MSCs	Increased expression of osteoblast-related markers such as osteopontin, RUNX2, and ALP Increased expression of miR-196a-5p and defective cell proliferation	[131,132]
Levetiracetam	Adipose and bone marrow derived MSCs Human Wharton’s jelly MSCs	Modulation of the release of inhibitory and excitatory neurotransmitters/regulation of the release of inflammatory factors such as bFGF, TNF-a, IL-6, and BDNF Regulation of the expression of antioxidant genes such as Cu/ZnSOD, signaling proteins such as PEBP1/regulation of the expression of genes related to apoptosis, survival and cell death such as BDNF/GDNF	[133,134]
Pregabalin	Bone marrow-derived MSCs	Decreased Notch1/p38-MAPK signaling pathway activity/ Reduction of the level of inflammatory markers such as TNF-α, NF-κB, p65, and IL-6/ Increased level of antioxidants	[135]
Gabapentin	Ovine-derived mesenchymal stem cells	Increased speed of mesenchymal cell division	[136]
Phenobarbital	TERA2.cl.SP12 stem cells	Induction of necrosis in neurons and reducing their differentiation	[137]
Carbamazepine	TERA2.cl.SP12 stem cells	Impairment of cell proliferation and reduced cell viability	[137]
Lamotrigine	TERA2.cl.SP12 stem cells	Induction of necrosis and reduction of cell viability	[137]

Studies have shown that VPA (10 mM for 6 h) activates the extracellular signal-regulated kinases (ERKs) and protein kinase B (AKT) signaling pathways in MSCs and causes their differentiation into hepatic cells. Both ERKs and AKT should be downstream targets of the HGF signaling pathway that are involved in the division, migration, and differentiation of stem cells. MSCs treated with VPA (1 mM for 24 h) showed increased expression of endodermal genes such as forkhead box A1/2 (FOXA1/2), SRY-box transcription factor 17 (SOX17), goosecoid, eomesodermin, tyrosine-protein kinase met, hepatocyte nuclear factor 1 homeobox B at the mRNA level, and FOXA2 and SOX17 at the protein level. Additionally, CXCR4 signaling increased in a dose-dependent manner, which seemed to be the result of increased hypermethylation of the CXCR4 gene promoter and chromatin remodeling. These results showed that VPA (2.5 mM for 3 months) might be involved in the differentiation of MSCs into hepatic cells through various mechanisms [115,116]. Moreover, exposure of MSCs obtained from elderly donors to specific concentrations of VPA increased their immortality by specifically activating the CXCR4 signaling pathway [117].

Recently, other studies evaluated the effect of VPA (5 mM for 24 h) on the differentiation of BMSCs into hepatic cells in 2D and 3D cultures [118]. Because VPA inhibits the activity of histone deacetylase, it increases the access of various transcription factors to chromatin and subsequently increases gene expression. After 21 days of cell culture, the presence of polygonal cells was observed in the culture medium, which is a characteristic of hepatic cells. Additionally, the mRNA expression of specific hepatic genes such as anti-albumin (ALB, a hepatic marker which reaches its highest level in mature cells), anti-α-1-fetoprotein (AFP, a marker of fetal hepatocytes and hepatoblasts), CK-18 (an intermediate filament protein of hepatocytes), and tyrosine aminotransferase (TAT, a marker of mature hepatocytes) were increased in both types of cell cultures, although such expression was higher in the 3D culture. These findings revealed that MSC differentiation into the hepatocytes might be facilitated in the presence of VPA, although these effects appeared to be concentration and time dependent.

In MSCs treated with VPA (5 mM for 48 h), a network of microRNAs plays a role in their differentiation into hepatocytes. The expression of miR-192-5p and miR-122-5p in these cells had a significant increase both in the early and more advanced days of differentiation. While the expression of miR-27b-3p, miR-23b-3p, and miR-24-1-5p did not change much in the early stages, they increased in the following days. These microRNAs, which are seen in mature liver phenotypes, play an important role in regulating the normal development and function of the liver. Moreover, in the early stages of hepatic differentiation, the levels of miR-26a-5p, miR-148a-3p, and miR-30a-5p showed a significant increase and remained elevated during hepatic differentiation. MiR-148a-3p inhibits epithelial-mesenchymal transition by silencing the Met/Snail signaling pathway. Additionally, miR-10a and miR-335 expression decreased in differentiated cells. To validate the results of this study, the expression of SRY-box transcription factor 11 (SOX11) and vimentin (VIM), which are the target genes of miR-122-5p, a highly regulated miRNA during hepatic differentiation, were investigated. MiR-122-5p negatively regulated the expression of both genes and caused hepatic differentiation and reduced levels of mesenchymal marker [119].

In AD-MSCs and BMSCs cultured in an osteogenic culture medium, VPA (0.5–3 mM) caused an increase in the expression of osteogenic genes, such as runt-related transcription factor (RUNX), BMP 2, cyclin-dependent kinase inhibitor 1 (p21WAF1), osterix and osteopontin, and improved their differentiation. However, such differentiation did not necessarily imply an increase in cell proliferation because VPA inhibited the proliferation of both cell types in a dose-dependent manner [9]. At variance, VPA (2.5 mM for 72 h) induced osteogenic differentiation by increasing the stability of cellular communication network factor 1 (CCN1) protein in MSCs [120].

In another study, the effect of VPA (1 mM) on the differentiation of MSCs derived from pancreatic islets into beta cells was investigated. Mature and functional cells expressed various genes, including NK6 homeobox 1 (NKX6.1), which is involved in β cell neogenesis. The expression levels of pancreatic and duodenal homeobox 1 and insulin 2 were lower in VPA-exposed cells, indicating that few cells had undergone advanced stages of differentiation. A significant decrease in glucose transporter 2 and paired box 6 mRNA expression was also observed, indicating that beta cells were not yet functionally differentiated. However, in the VPA group treated with glucose, the highest expression of genes related to the differentiation of beta cells was observed, which indicates that this combined treatment can cause the production of mature beta cells, even if they were not yet functional. Additionally, the number of positive insulin cells was significantly higher in the groups treated with VPA. Therefore, insulin secretion was higher in the VPA-treated groups in parallel with the greater differentiation of β cells [121].

According to the change of the medium, MSCs exposed to VPA (1 mM) can also differentiate into cardiac cells. Indeed, cultured VPA-exposed AD-MSCs in a fibrin scaffold after 4 weeks of incubation showed a high percentage of expression of several cardiac primary transcription genes (NK2 homeobox 5, NKX2.5; heart and neural crest derivatives expressed 2, HAND2; heart and neural crest derivatives expressed 1, HAND1, and GATA Binding Protein 4, GATA4) and some structural genes (connexin 43, Cx43; β-Myosin Heavy Chain, βMHC; cTnI, cardiac troponin I; and myosin light chain 2, MLC2v). These genes have a special role in heart growth, differentiation, and function. Additionally, immunofluorescence staining studies for Cx43 showed that gap junctions were formed between the differentiated cells, which was another reason for the differentiation of these cells into cardiac cells [122].

Notably, the differentiation of MSCs into neuron-like cells was made possible by using a composite 3D scaffold combined with VPA (1 mM for 21 days) induction [123]. Indeed, MSCs moved towards neurization by expressing neuronal markers, such as nestin and neuro-D1. Moreover, the length of neurites was significantly increased in the treated cells. This was consistent with an increased expression of the microtubule-associated protein 2 (MAP-2) gene, which is known to be a dendritic marker. The expression of potassium voltage-gated channel subfamily H member 2/5 (KCNH2/5) genes and markers of neuronal-specific calcium channels also showed a significant increase compared to the control group [124]. Moreover, placenta-derived MSCs exposed to VPA were differentiated into neural cell lines in a neural medium. Such induction was possible through the reduction of bone morphogenetic protein 2 (BMP 2) expression. At variance, the expression of bone morphogenetic protein 4 (BMP 4), which is an inhibitor of glycogen synthase kinase 3β, showed a significant increase. In turn, the increase in BMP 4 expression caused the activation of the Wnt/β-catenin signaling pathway, which resulted in the differentiation of placenta-derived MSCs into neurons. In addition, Notch 1 signaling, which mainly plays a role in maintaining the mesenchymal state of cells and prevents their final differentiation, showed a significant decrease. In general, it was reported that the exposure to VPA (0.5 mM) induced a decrease in the expression of early neural genes and an increase in the expression of mature neural markers [125]. Moreover, the reduction of cell divisions in MSCs exposed to VPA could also be a sign of cell aging. Although mesenchymal cells exposed to VPA (10 mM for 3 days) did not increase senescence markers, such as senescence-associated beta-galactosidase (SA-β-gal), they could prevent cell proliferation by inhibiting the cell cycle in the G2/M phase [126].

Pretreatment of MSCs with VPA in some diseases also showed significant therapeutic results. For example, in 2016, Linares et al. evaluated preconditioned MSCs with VPA (2.5 mM) before intranasal injection of them into the brain of a transgenic mouse model of Huntington’s disease. This genetic manipulation increased the expression of genes involved in energy metabolisms such as peroxisome proliferator-activated receptor-gamma coactivator (PGC)-1α, cytochrome C oxidase subunit 6B2 (Cox6b2), and ATPase H+/K+ transporting non-gastric alpha2 subunit (Atp12a) in the treatment groups. Increased expression of genes involved in antioxidant defense pathways such as serpin family B member 1 (Serpinb1b), glutathione peroxidase 6 (Gpx6), and metallothionein 2 (Mt2) showed that these cells have become resistant to oxidative stress. Additionally, heat shock protein 27 (Hsp27), Hox, and Hsp A1l genes’ expression, which are involved in cellular stress pathways, and Erc1, Naip1, and Fas apoptotic inhibitory molecule 2 (Faim2) genes, which are anti-apoptotic markers, were significantly increased. Preconditioned MSCs showed elevated expression of fibroblast growth factor 15 (FGF-15), FGF-21, neuron derived neurotrophic factor (NDNF), growth differentiation factor 1 (GDF-1), bone morphogenetic protein 3 (BMP 3), and neurotrophin 3 (NTF3) which are growth and trophic factors. Elevated levels of bone morphogenetic protein 6 (BMP 6) and interleukin 19 (IL-19), which are anti-inflammatory cytokines, were also observed, which shows that an anti-inflammatory environment has been created in the brain after this treatment [127]. In addition, pretreatment of MSCs with VPA (1 mM for 6 days) induces apoptosis in T cells and has anti-proliferative effects on these cells. It has been shown that VPA can be considered as an effective treatment regimen in graft versus host disease [128]. 

In a stroke model of rats, it was shown that pretreatment of MSCs with VPA (2.5 mM for 3 h) and lithium increase their homing ability toward the infarcted areas. In addition, the volume of the infarct decreased, the function of the damaged areas was partially restored, and angiogenesis occurred in some areas of the striatum and cortex. Gene expression studies showed that the strengthening of CXCR4 and matrix metallopeptidase 9 signaling that occurs under the influence of VPA and lithium plays a role in mediating the effects of these compounds. However, after transplantation, most of the cells continued to express mesenchymal markers such as fibronectin and intercellular adhesion molecule 1 (CD54), and a few of them differentiated into endothelial cells or astrocytes, which indicates that VPA or lithium are not very effective on the differentiation ability of MSCs in ischemic brain [129].

In a gene therapy study that used MSCs to transfer the herpes simplex virus type 1 thymidine kinase gene to intracranial glioma, it was shown that in the presence of different concentrations of VPA (0–4 mM), the expression of Cx43/26 genes, tumor suppressor genes, increased in a dose-dependent manner and caused the death of target cells. Moreover, treated cells had increased gap junctions that let them transfer toxic metabolites to the adjacent cells and induce apoptosis. In this regard, it was observed that apoptotic bodies were significantly reduced in the treatment groups. Histological evidence also showed that the size of the tumor in groups treated with MSCs-TK/VPA was significantly reduced compared to the control group [130].

### 5.2. The Effect of Phenytoin on MSCs

The osteogenic potential of phenytoin was investigated in dental pulp MSCs. Isolated MSCs were treated in a culture medium containing different concentrations of phenytoin (12.5, 25, 100, and 200 µM for 10 days). The results suggested that phenytoin increased osteoblast-related markers, such as osteopontin and RUNX2, in MSCs [131]. Notably, a study revealed that phenytoin leads to cell proliferation defects in mouse embryonic lip MSCs. Indeed, phenytoin (50 μg/mL for 3 days) seemed to upregulate miR-196a-5p, which subsequently contributed to the development of cleft lip disease [132].

### 5.3. The Effect of Levetiracetam (LEV) on MSCs

Recently, the therapeutic potential of AD-MSCs and BM-MSCs alone or simultaneously in combination with levetiracetam (LEV) was investigated in a rat model of epilepsy. In this study, seizures were associated with decreased levels of GABA and increased levels of dopamine, glutamate, bFGF, TNF-α, IL-6 and BDNF. Both types of MSCs quelled the behavioral symptoms and biochemical changes caused by epilepsy and improved the release of neurotransmitters. Additionally, these effects increased when each of these treatments was accompanied by LEV (300 mg/kg). In fact, this combination therapy synergistically caused brain tissue restoration and biochemical changes [133]. 

The combined treatment of LEV and MSCs was also used to treat cerebral ischemia. Based on the reported findings, the neuroprotective effects of LEV were mainly through binding to SV2A, which then bound to synaptotagmin and regulated calcium-dependent exocytosis of synaptic vesicles. It also elevated the expression of BDNF, GDNF, copper- and zinc-containing superoxide dismutase (Cu/ZnSOD), an antioxidant gene, and phosphatidylethanolamide binding protein 1 (PEBP1), a signaling protein. As evidenced previously, BDNF signaling is involved in neuronal survival, cognitive functions, the reduction of intracellular calcium accumulation and the inhibition of apoptosis. GDNF protects neurons from cell death induced by ischemic injury and excitotoxicity. PEBP inhibits the mitogen-activated protein kinase (MAPK) signaling pathway and exerts its effects by reducing microglial excessive activation and promoting neuronal survival. Cu/ZnSOD is also involved in the reduction of neuronal death. Since the combined treatment of LEV (500 mg/kg) and MSCs had a better outcome in terms of reducing infarct volume and improving memory functions, it seems that this effect was caused by the strengthening of brain defense systems in the treated rats [134]. 

### 5.4. The Effect of Pregabalin on MSCs

A study investigated the modulatory effect of BMSCs on pregabalin (30 mg/kg/day, per os) treatment in paclitaxel (PTX)-induced peripheral neuropathy. In the groups treated with PTX, increased phosphorylation and subsequent activation of the notch receptor 1 (Notch1) and p38-MAPK signaling pathways were observed. As the results showed, the therapeutic potential of pregabalin and MSCs used simultaneously translated into an anti-inflammatory effect by reducing the activity of these pathways and their downstream, such as TNF-α, NF-κB, p65, and IL-6, and increasing antioxidant markers. Additionally, in the treated mice, the lesion of the sciatic nerve was significantly reduced, and the axons and myelin sheath were similar to those in the control group [135]. 

### 5.5. The Effect of Gabapentin on MSCs

It has been found that gabapentin (1 nmol/L) and its derivatives increase the rate of MSC proliferation. According to the culture medium, these cells can differentiate into chondrocytes, adipocytes and osteoblasts. In fact, gabapentin and its analogs can act as a stimulator of cell division and differentiation [136]. 

## 6. Human Stem Cell Lines and Risk of Developmental Neurotoxicity with ASMs

In a comprehensive study conducted by William et al. in 2015, the effects of 4 ASMs on stem cell lines were investigated to assess the risk of toxicity, cell differentiation, cell cycle and viability. According to their reports, transient exposure of stem cells to phenobarbital reduced the differentiation rate and production of new neurons through the induction of necrosis. VPA increased cell death by increasing DNA fragmentation in both differentiated and undifferentiated stem cells. It also reduced the proportion of cells in S and G2/M phases and as expected, the rate of cell divisions decreased. Carbamazepine diminished cell viability by increasing DNA fragmentation, and in high concentrations, it caused disruption in the proliferation. Lamotrigine did not have specific effects on the cell cycle, but it decreased cell viability through the induction of necrosis [137].

## 7. Limitations of MSCs

Although MSCs have shown high therapeutic potential, they also have some limitations. For example, the sensitivity of the techniques used, differences in sampling methods, the method of sample manipulation, the existence of heterogeneity of epilepsy in terms of the cause and type of the disease and the small size of the study groups could have affected their functions [8,138]. MSCs function may also be neutralized by the rapid clearance from the body or because of the immune system’s reaction against them [85]. Since the amount of a patient’s own stem cells is limited, these cells are mainly maintained in a proliferative state. The overgrowth of cells in confined space and reduced access to other supporting factors may cause additional stress on the cells. Furthermore, their removal from niche due to the use of anesthetics might negatively impact their viability and quantity [57,67,89]. It is worth noting that undifferentiated, dividing MSCs, like other stem cells, are highly tumorigenic [139].

In addition to the limitations of retention of these cells in the CNS, they sometimes show little tendency to migrate [63,140]. Transplanted cells produce and release various molecules that can be further regulated by the pathological environment [63]. The effects of them can also be limited by the reactions of the host body and regulated by a number of factors that should be further investigated in future studies. Moreover, most studies have been performed mainly on animal specimens and on a low number of specimens, so more detailed studies with a larger number of specimens should be performed before conducting studies on human specimens [55,65,141]. Drug interactions and synergistic effects with MSCs and changes in the function of injected cells in patients are also important issues that should be considered in future research. Finally, these cells must be examined over a long period of time after transplantation to reveal their long-term effects [61]. 

All these concerns are further complicated by the interaction of stem cells with ASMs, which could be detrimental to the success of this innovative therapeutic intervention. Studies in models are still limited, but potentially showed a reduction in the effectiveness of stem cell transplantation in presence of specific ASMs. This suggests that drug therapy should be modified in that case, so that stem cells could be tested as a possible therapeutic intervention.

## 8. Ethical Issues

The ethical implications of stem cell therapeutic use are at different levels and have been recently reviewed by other investigators, who should be referred to deepen this topic [142]. Like any cell, iPSCs derived from any individual will inherently contain a vast amount of private information (DNA) which, if used carelessly, may violate the law, morality and the privacy of individuals. Even if the starting cell donor is not alive, the iPSCs contain his/her close relatives’ information, hence potentially bringing about ethical and legal challenges related to individual privacy [143]. Furthermore, pluripotent stem cell lines can be derived from the inner cell mass of the 5- to 7-day-old blastocyst. However, human embryonic stem cell (hESC) research is ethically and politically controversial because it involves the destruction of human embryos [24]. The ethical dilemma involving the destruction of a human embryo was and remains a major factor that may have limited the development of hESC-based clinical therapies [29]. The advantage of using MSCs is mainly that they overcome the need to destroy embryos in order to recover stem cells. This practice has been abolished in different countries, and only commercial cell lines obtained by embryos prior to the adoption of strict rules could be available [144]. However, the use of MSCs also poses ethical issues.

A major problem with the ethical implications is in the need to perform reproducible experiments at both preclinical and clinical levels [145]. When using MSCs, reproducibility is made problematic because of the scarce adherence of investigators to the shared definition of these cells as established by the International Society for Cell & Gene Therapy (ISCT) [146]. This is an important source of variability which makes it very problematic to establish how the different characteristics of MSCs, used by the investigators in performing their experiments, could have impacted on the obtained results. Indeed, reproducibility and transparency are strongly affected by this lack of adherence to consensus, making the translatability of preclinical findings to possible therapeutic approaches ethically questionable [145].

It also has to be mentioned that MSCs can be obtained from different sources, but this richness in opportunities to obtain them represents a problem because of the heterogeneity of cells obtained for therapy; there is a particular problem concerning their ability to proliferate, differentiate and express different surface markers. This also makes the adherence to ISCT recommendations to identify MSCs problematic [147]. Non-invasive procedures could be preferable in comparison to others involving, for instance, bone marrow or adipose tissue as sources of MSCs, and the obvious ethical implications of procedures required to obtain them. Lastly, the patient age is another issue: age is a strong determinant of the possibility to obtain efficient MSCs and doubts could be raised on the motivations of proposing MSCs to patients who could not receive such clear beneficial effects from this approach [147]. Although the clinical application of MSCs has shown beneficial effects in the therapy of autoimmune and chronic inflammatory diseases, their ability to promote tumor growth and metastasis and the overestimated therapeutic potential of MSCs still provide concerns for the field of regenerative medicine [148,149,150].

## 9. Potential Risks and Adverse Effects

It should be noted that, according to the route of their administration, therapeutic potentials can be different. Intracerebral injection allows for more direct cell homing; however, local injection causes the poor distribution of the injected cells, and, as an invasive method, it can cause bleeding and even induce seizures. However, the intraarterial injection causes a better distribution of stem cells, but in some cases, it has been reported that they could not cross the blood–brain barrier, and in some cases, they adhere together and aggravate brain damage by forming microemboli [151,152,153].

IV injection, like intra-arterial injection, is a less invasive method, but like intra-arterial injection, cells may face problems crossing the blood–brain barrier. Or they may migrate to the perivascular areas of other organs and increase the risk of ectopic growth or secrete various factors in other organs. Another important limitation of this type of injection is the pulmonary first-pass effect, which leads to a large number of these cells being out of reach, which in turn requires increasing the injection dose and reducing their effectiveness [154,155]. In contrast, intrathecal injection of stem cells has been used in many types of neurological diseases [151], and no specific side effects have been observed in these patients. This method appears to be safer and results in a dynamic flow and good distribution of stem cells in brain areas [151,156].

In general, the adverse effects of stem cells include the induction of some angiogenic diseases, arteriosclerosis, increased restenosis, aberrant calcification, arrhythmias, and teratoma formation, which must be considered in future research [157]. Therefore, toxicological evaluation of stem cells, investigation of the relationship between concentration and toxicity, and possible judicial interference are among the things that can affect their efficiency [158].

## 10. Conclusions

Recent advances in MSCs isolation and manipulation for therapeutic purposes have revolutionized the field of regenerative medicine and cell therapy. Isolation of these cells from allogeneic and autologous sources and then transplantation to the host body have shown enormous therapeutic potential in a variety of epilepsy models. In addition to their unique properties in tissue regeneration, these cells might also restore its normal properties. The observations described here also well demonstrate the ability of these cells to address epilepsy and overcome several limitations of current therapies. Such effects are due to (1) their high homing ability in the host tissue and coordination with the nervous system; (2) their relative high self-regenerative ability over a long period of time; (3) the release of a variety of trophic and immunity factors, in addition to different neuromodulators and neurotransmitter; (4) their ability to deliver therapeutic molecules, which has made them reliable therapeutic tools. Overall, treatments with MSCs are associated with enhanced seizure threshold, shortened seizure duration and reduced frequency and amplitude of epileptic discharges. Despite their high therapeutic potential, these cells also have limitations. Therefore, in order that we might use them more efficiently in clinical applications, more detailed studies are needed in the future.

## Figures and Tables

**Table 1:** Stem cells’ classification.

**Stem Cells**	1-Allogenic adult stem cells, derived from another person’s specialized tissue; have ethical concern 2-Autologous adult stem cells, derived from the individual’s own specialized tissue; without ethical concern	Embryonic stem cells	**Origin**	**Characteristic**	**Types**	**Ethical Issues**	**Ref**
			Derived from an embryo in the first four blastomeric cleavage phases	Totipotent	-	Destruction of embryos Creation of embryos specifically for research purposes Payment to oocyte donors Medical risks of oocyte retrieval Protecting reproductive interests of women in infertility treatment [24] Social impact: (a) Desensitization to the destruction of human life with value [25] (b) Contributions to social oppression [26]	[27]
			Derived from an embryo after the fourth blastomeric cleavage phase	Pluripotent	Embryonic stem cell		[28]
		Adult stem cells	Mesenchymal stem cells (MSCs)	Multipotent	Umbilical cord-derived mesenchymal stem cells, bone marrow, and adipose-derived mesenchymal stem cells	Since the use of these cells does not require the destruction of the embryo, it brings fewer ethical problems. However, as allogenic stem cells contain donor DNA, this may be associated with safety, legal and ethical challenges associated with the privacy of individuals. Autologous stem cells do not pose a particular ethical problem and do not interfere with the recipient’s immune system [29,30]	[31,32,33]
			Induced pluripotent stem cells	Pluripotent	Fibroblasts, dental pulp, deciduous teeth, renal epithelial cells, and urine		[34,35,36]
			Neural stem cells. Derived out of the periventricular sub-ependymallayer and sub-granular zone of the dentate gyrus	Multipotent	Glia (oligodendrocyte precursor cells) and neurons		[37,38]
			Hematopoietic stem cells	Multipotent	Lymphoid progenitor cells, hemangioblasts, peripheral blood stem cells, myeloid progenitor cells		[39,40,41,42]
			Myoblasts	Multipotent	Cardiac, skeletal, smooth muscle cells		[43,44,45]

**Table 2:** MSCs transplantation studies in animal models of seizure/epilepsy.

Model	Type of Stem Cell	Injection Method	Volume Concentration	Measured Parameters	Findings	Ref
Pilocarpine induced SE rats	hUC-MSCs	Bilateral intra hippocampus	10^5^ cells	Hippocampal morphology/inhibitory transmission/epileptic properties	Recovery of hippocampal and GABAergic neurons/Reduced duration and incidence of epilepsy/Maintenance of neuronal circuits’ integrity/Attenuation of MFS and glutamate toxicity/Expression of various cytokines	[58]
Pilocarpine-lithium induced SE rats	hUCBC	Tail vein	1 × 10^6^ cells/rat	Frequency and duration of SRS/hippocampal neuronal densities	Both frequency and duration of SRS were decreased/elevated neuronal densities in the hippocampus	[59]
PTZ-induced epileptic rats	hUCB-MSCs	Tail vein	10^6^ MSCs/rat	Cognitive and motor function/seizure activity/oxidant and antioxidant measurements/GABA level determination	Decreased oxidative stress impairments and cognitive and motor dysfunction/enhanced GABAergic circuits	[60]
Lithium- pilocarpine induced SE rats	hUCB-MSCs	Intra right hippocampus	5 × 10^5^/2 µL	Hippocampal volume/inflammatory changes/hippocampal glucose metabolism	Elevated hippocampal glucose metabolism/bilaterally migration of cell in hippocampi	[61]
Pilocarpine induced SE mice	MSC-EVs	In vitro/In vivo; Intravenous	50 μg MSC-EVs diluted in 150 μL sterile PBS	Anti-oxidative function/SE activity/neural function and morphology	Antioxidant activity of MSC-EVs by Nrf2 signaling/restore structural alterations and neuronal dysfunction/learning, memory and SE improvement	[62]
Human model of drug-resistant epilepsy	Autologous BMSCs Neuro-induced MSCs	Intravenous	2–5 × 10^6^ cells/mL 2.7–8 × 10^6^ cells/mL	Epileptic activity/cognitive function	Cognitive improvements/immunoregulatory effects	[63]
Lithium- pilocarpine induced SE rat	Autologous BMSCs	Intravenous	1.0 × 10^6^ cells/mL	Seizure frequency/ cognitive function	Reduced neuronal cell death/Inhibition of aberrant MFS/Cognitive function improvement/Attenuation of epileptogenesis	[64]
Kainic acid induced SE rat	BMSCs	Bilateral intraventral hippocampus	20,000 cell in 1.5 μL PBS	Epileptic activity	MSC transplant did not change the duration and severity of seizure	[65]
Kindling epilepsy rats	Autologous BMSCs	Unilateral intrahippocampal	2 μL	Adenosine receptors expression	Bidirectional change in adenosine receptors/Maintenance of the balance of adenosine receptors	[66]
Kindling epilepsy rats	ESCs	Lateral brain ventricles	-	Seizure activity	Prevention of seizure activity	[67]
Kainic acid induced epileptic mouse	hMSCs	Infrahippocampal	2.5 μL	Seizure activity	hMSCs worked as a vehicle to deliver adenosine/ Improvement of seizure activity	[68]
Kainic acid induced epileptic rats	hMSCs	Left hippocampus	5 μL	Brain injury/ hippocampal morphology/epileptic activity	Reduced seizure duration and hippocampal neural loss/Amelioration of seizures/Reduced apoptosis	[69]
Pilocarpine induced epileptic rats	BMSCs	Intravenous	500 mL	Histological and morphological analysis/	Functional and structural improvements of hippocampus	[70]
Lithium-induced SE rats	BMCs	Intravenous	1 × 10^7^ cells/mL	Epileptic activity/morphological change/LTP	Lower duration and frequency/ suppressed seizure/Decreased neural lose/Prevention of spontaneous seizures/Reduction of cell loss and hippocampal change/Improvement of LTP formation	[71]
Pilocarpine induced epileptic rats	BMCs	Intravenous	1 × 10^7^ cells/mL in a volume of 100 μL	Seizure activity/ inflammatory and anti-inflammatory cytokines/	Seizure duration and frequency reduction/Decreased pro-inflammatory cytokines/Elevation of anti-inflammatory cytokine	[72]
Lithium chloride pilocarpine induced epileptic rats	BMSCs	Right lateral ventricle	5 × 10^6^ cells	GABAergic transmission/epileptic activity	Hes1 silencing caused BMSCs to differentiate into GABAergic neuron	[73]
Pilocarpine induced epileptic rats	BMSCs	lateral ventricle	5 × 10^6^ cells	Epileptic activity/cell migration and differentiation	Rate of mortality reduction/SRS frequency and epileptic activity decreased/GABAergic neurons increased	[74]
Pilocarpine induced SRS rats	BMMCs	Tail vein	1 × 10^7^ cells	Expression levels of GDNF, NGF, BDNF, TGF-β1, and VEGF, and their receptors	Upregulation of trophic and growth factors/Neuroprotective effects	[75]
PTZ-induced epileptic seizures rats	BMSCs	Intravenous	3 × 10^6^ cells/rat	The level of excitatory and inhibitory neurotransmitters/oxidative and anti-oxidative factors/HPA axis hormones	Reduction in epileptic activity by balancing between inhibitory and excitatory neurotransmitters/ Anti-oxidant activity/Modulation of sex hormonal profile and inflammatory response	[76]
Pilocarpine induced epileptic rats	BMSCs	Intravenous	2 × 10^7^ cells	Excitatory and inhibitory neurotransmitters concentration/oxidation and anti-oxidation activity/immunomodulatory factors	Reduction in oxidative functions and lipid peroxidation/Downregulation of inflammatory cytokines/Upregulation of anti-inflammatory cytokines and IGF-R signaling/Reduced excitation in hippocampus	[77]
Pilocarpine induced epileptic rats	BMMCs	Intravenous	1 × 10^7^ cells	Cytokine production/epileptic activity	Reduced inflammatory cytokines/Increased anti-inflammatory cytokines	[78]
Pilocarpine induced epileptic rats	BM-MSCs AD-MSCs	Tail vein	100 µL	HSP-70, S100β, and caspase-8 levels/TLR-4/BBB integrity	Decline in the level of S100β, HSP-70, and caspase-8, and TLR-4 gene expression/Neuroprotection/Reduced neural loss	[79]
Temporal lobe epilepsy patients	BMMC	Intra-arterial	15 mL 1.52 × 10^8^ to 10 × 10^8^ cells	MRI/EEG/verbal and nonverbal memory	Better memory performance/Decreased epileptic spikes	[80]
Lithium–pilocarpine induced-SE rats	BMMCs	Intravenous	1×10^7^ cells in 200 μL	SRS activity/cognitive function	Decline in frequency of seizures/Improved long-term spatial memory and learning	[81]
Kainic acid induced epileptic rat	ADSC	Intra left hippocampus	50,000 cells	Neuronal cell markers/learning and memory/neurotrophins/apoptotic and anti-apoptotic factors	Elevated anti-apoptotic and neuronal cell marker expression/Learning and memory improvement	[82]
Pilocarpine induced epileptic mice	ADSC	Intraperitoneal	40 mg/kg	BBB leakage/SRS/cognitive functions	Attenuation of seizure spikes/Reduced BBB leakage	[83]
Convulsive seizure induction by maximum electroshock, mice model of seizure	MCAT	Intrahippocampal	1 × 10^5^ cells (50,000 per hemisphere)	Seizure duration and activity/mortality rate/inflammatory and anti-inflammatory markers	Anticonvulsant effects/Downregulation of inflammatory markers	[84]

**Table 3:** MSCs transplantation studies in human models of seizure/epilepsy.

Model	Type of Stem Cell	Injection Method	Volume Concentration	Measured Parameters	Findings	Ref
DS patient- derived induced pluripotent stem cells	hUC-MSCs	In vitro	-	Oxidative stress markers/Ca 2+ levels/Inflammation	Increased anti-oxidative enzymes: GSH, SOD1/2, and GPX and reduced oxidative markers: MDA, Ca 2+ and ROS/Elevated anti-inflammatory factors: TGF-β and IL-10/6 and lowered interleukin-1 and TNF-α	[85]
Drug-resistant epilepsy patients	Autologous BMSCs	Intravenous injection followed by intrathecal injection	1.0–1.5 × 10^6^ cells/kg/f 0.1 × 10^6^ cells/kg, respectively	Epileptic activity/Seizure frequency/Depression and anxiety	Decreased seizure frequency and paroxysmal activity/Reduced depression and anxiety score	[8]
Drug-resistant epilepsy patients	BMNCs	Intrathecal BMNCs: 0.5 × 10^9^; intravenous: 0.38 × 10^9^–1.72 × 10^9^ BMMSCs: 18.5 × 10^6^–40 × 10^6^	-	Cognitive function/ Epileptic seizure	Cognitive and neurological improvement	[86]
Autoimmune refractory epilepsy patients	Autologous ADRCs	Intrathecal 3 times every 3 months	4 mL	Cognitive function/Epileptic effects/Inflammatory and anti-inflammatory markers	Elevated level of anti-inflammatory cytokines/Reduced epileptic activity/Cognitive function improvement	[87]

## Data Availability

Data sharing is not applicable to this article as no datasets were generated or analyzed during the current study.
